# Single-Vendor Electronic Health Record Use Is Associated With Greater Opportunities for Organizational and Clinical Care Improvements

**DOI:** 10.1016/j.mayocpiqo.2022.05.001

**Published:** 2022-05-31

**Authors:** Hanadi Y. Hamadi, Shehzad K. Niazi, Mei Zhao, Aaron Spaulding

**Affiliations:** a Department of Health Administration, University of North Florida, Jacksonville, FL; b Department of Psychiatry and Psychology, Robert D. and Patricia E. Kern Center for the Science of Health Care Delivery, Mayo Clinic, Jacksonville, FL; c Division of Health Care Delivery, Robert D. and Patricia E. Kern Center for the Science of Health Care Delivery, Mayo Clinic, Jacksonville, FL

**Keywords:** AHA, American Hospital Association, EHR, Electronic Health Record, HHI, Herfindahl–Hirschman Index, IT, Information Technology, QI, quality improvement, RRR, relative risk ratio, VHA, Veterans Health Affairs

## Abstract

**Objective:**

To compare how hospitals that use single-vendor vs best-of-breed electronic health record (EHR) vendors utilize clinical and organizational evaluation capabilities.

**Methods:**

Data from the 2018 (June 1, 2016, to December 31, 2017) American Hospital Association Information Technology Supplement Survey and Medicare Final Rule Standardizing File were used. Multinomial logistic regression analysis of hospitals (n=1902) was conducted to identify hospital characteristics associated with the use of EHRs for (1) clinical care evaluation capabilities and (2) organizational evaluation capabilities.

**Results:**

Single-vendor EHR hospitals were more likely (relative risk ratio, 3.37; 95% confidence interval, 1.97-5.76) to use EHRs for clinical care and organizational evaluation capabilities. Not-for-profit hospitals were more likely to use EHRs for all organizational evaluation capabilities than government nonfederal hospitals. For-profit hospitals were less likely to use EHRs for organizational or clinical evaluation capabilities than government nonfederal hospitals.

**Conclusion:**

Hospitals using the single-vendor EHR system were more likely to engage in clinical care and organizational evaluation than hospitals using best-of-breed EHR systems.

Hospitals and hospital systems increasingly use electronic health record (EHR) systems to support quality improvement (QI) efforts, monitor patient safety, measure organization performance, identify high-risk patients, and improve administrative and clinical processes.^1^, ^2^, ^3^ One common theme across organizational responses is that a greater number of health care delivery systems are addressing resultant or preexisting fragmentation and lack of interoperability in health information technology (IT) through investment in single-vendor as opposed to best-of-breed solutions. A single-vendor solution occurs when the hospital uses the same vendor for all of its EHR needs, whereas a best-of-breed solution is when the hospital integrates the best EHR components from multiple vendors.^4^, ^5^, ^6^ In either case, the solution chosen is focused on how the hospital can best leverage the chosen strategy to reduce costs and improve value-added care.^7^^,^^8^ In many hospitals and health systems, the installation of a new EHR has prompted a shift to a single vendor. For example, over the past decade, health systems have spent considerable financial sums to convert to single-vendor EHR systems, including Mayo Clinic, which reports spending $1.5 billion, and the Kaiser Permanente, spending $4 billion to convert to Epic.^9^^,^^10^ With these vast expenditures, organizations have to believe that there are benefits to pursuing this single-vendor EHR strategy that would otherwise not be achievable or financially viable when considering the best-of-breed solutions.

Although vendors have built a broad range of technologies to facilitate increased value-added care and facilitate patient engagement, wide variation in the adoption of these capabilities by health care organizations exists. This variation has led to questions concerning how hospitals use their EHR when considering best-of-breed vs single-vendor EHRs. Thus, understanding how hospitals are currently using their EHR data is essential both as (1) policy initiatives seek to incentivize hospitals to use their EHR data for performance and population health management and (2) organizations seek to continue to improve both clinical and organizational performance through better leveraging data collected through the EHR. As a result, this study examines how organizations use the EHR capabilities for clinical and organizational evaluation. Specifically, we hypothesize that hospitals with a single-vendor EHR system will be better able to use clinical and organizational evaluation tools.

## Methods

### Data Source

All hospitals that completed the American Hospital Association (AHA) IT Supplemental from 2018 which includes data from June 1, 2016, to December 31, 2017, were included in the study. The AHA IT survey queries hospitals concerning their EHRs, health information exchange, IT vendors, and how hospitals use data collected through electronic systems.^11^ Additionally, hospital characteristics were collected from the AHA annual survey, which includes data concerning hospital type, size, and services offered and boasts at least a 75% completion rate.^11^ Finally, the Medicare Final Rule Standardizing File was used to collect the case mix index and disproportionate share for hospitals within the study.^12^

### Measures: EHR Evaluation Capabilities

Within the AHA IT supplement, hospitals were asked to select all that apply on whether they have used electronic clinical data from the EHR or other electronic system in the hospital to (1) create a dashboard with measures of organizational performance, (2) create a dashboard with measures of unit-level performance, (3) create individual provider performance profiles, (4) create an approach for clinicians to query the data, (5) assess adherence to clinical practice guidelines, (6) identify care gaps for specific patient populations, (7) support a continuous QI process, (8) monitor patient safety (eg, adverse drug events), (9) identify high-risk patients for follow-up care using an algorithm or other tools, or (10) none of the above.

We grouped EHR capabilities into 2 categories; those that focus on organizational evaluation (capability, 1-4) and those that focus on clinical care evaluation (capability, 5-9). We then created 3 ordinal count outcome variables. The first outcome variable was hospitals that used their electronic clinical data from the EHR for organizational evaluation (none = 0 capability, some = 1-3 capabilities, and all = 4 capabilities). The second was hospitals that used their electronic clinical data from the EHR for clinical care evaluation (none = 0 capability, some = 1-4 capabilities, and all = 5 capabilities). Finally, hospitals used their electronic clinical data from the EHR for clinical care and organizational evaluation (none = 0 capability, some = 1-7 capabilities, and all = 9 capabilities).

### Measures: Hospital Characteristics

On the basis of the literature, we identified key hospital characteristics that have impacted hospital performance and EHR use. Same or single-vendor EHR environments were identified using the AHA IT survey and operationalized as a binary variable indicating the same EHR vendor was used for inpatient and outpatient care (yes/no). Hospital size is defined as the total number of staffed inpatient beds.^13^ Hospital ownership is reported as government nonfederal, for-profit, and not-for-profit.^14^ Teaching hospital status is recorded as teaching vs nonteaching.^15^ Hospital system membership indicates system members vs a stand-alone facility. The location of hospitals is operationalized by indication of urban (0) vs rural (1) and region (West, Midwest, South, or Northeast). A disproportionate share identifies hospitals serving a large volume of patients who are uninsured or on Medicaid. Similarly, the case mix index is used to control for disease severity at the hospital level. Average Medicare and Medicaid percentages are measured as the proportion of a hospital’s total Medicare or Medicaid visits and total inpatient admissions.^16^ Finally, market competition for hospitals was measured using the Herfindahl–Hirschman Index (HHI), in which an HHI of 0 indicates a competitive market.^17^^,^^18^

### Statistical Analyses

The study population was described by means and counts. Continuous variables were assessed by the Kruskal–Wallis test and categorical variables by the Pearson χ^2^ test. Multinomial logistic regression analysis assessed the associations between hospital characteristics and EHR capabilities.^19^ Three separate models were used. Model 1 assessed the use of clinical care evaluation components. Model 2 assessed the use of Organizational Evaluation components, and Model 3 assessed both clinical care and organizational evaluation components. Pairwise deletion was used for missing data, and the data set was reviewed for extreme values that might bias the analysis. STATA 16 was used to run all analyses, and models were estimated through maximum likelihood. Relative risk ratios (RRRs), standard errors, and 95% CIs are reported.

## Results

There were a total of 1902 hospitals in our sample, of which 1384 (73%) reported having a single-vendor solution, and 518 (27%) used a best-of-breed solution. Most hospitals have at least 1 EHR capability for either clinical or organizational evaluation (95.74%). Our descriptive analysis (Table 1) reports that 55.89% of hospitals use all EHR clinical care evaluation capabilities, whereas 58.31% use all organizational evaluation capabilities and 46.85% use both clinical and organizational evaluation capabilities.

**Table 1:** Description of Electronic Health Records (EHR) Clinical Care, Organizational, or Both Evaluation Capabilities by Categorical Hospital Characteristics, 2018

	Clinical care capabilities							Organizational evaluation capabilities							All capabilities: clinical and organizational evaluation						
	None		1-4		All		*P* value	None		1-3		All		*P* value	None		1-7		All		*P* value
	N	%	N	%	N	%		N	%	N	%	N	%		N	%	N	%	N	%	
Categorical, n																					
Single-vendor EHR							<.001							<.001							<.001
No	160	31	161	45	197	55		42	8	243	51	233	49		32	6	333	69	153	31	
Yes	248	18	270	24	866	76		59	4	449	34	876	66		49	4	597	45	738	55	
Ownership														<.001							<.001
Government nonfederal	99	35	71	38	114	62		29	10	103	40	152	60		25	9	157	61	102	39	
For-profit	65	23	27	13	186	87		23	8	68	27	187	73		17	6	92	35	169	65	
Not-for-profit	244	18	333	30	763	70		49	4	521	40	770	60		39	3	681	52	620	48	
Member of system							<.001							<.001							<.001
No	172	38	126	46	150	54		55	12	208	53	185	47		47	10	284	71	117	29	
Yes	236	16	305	25	913	75		46	3	484	34	924	66		34	2	646	45	774	55	
Region							0.008							0.489							0.255
West	67	20	87	33	180	67		19	6	123	39	192	61		16	5	172	54	146	46	
Midwest	97	20	92	24	292	76		19	4	169	37	293	63		16	3	220	47	245	53	
South	189	25	170	30	400	70		43	6	290	40	426	59		34	4	387	53	338	47	
Northeast	55	17	82	30	191	70		20	6	110	36	198	64		15	5	151	48	162	52	
Teaching							<.001							<.001							<.001
No	213	28	192	34	367	66		64	8	304	43	404	57		55	7	407	57	310	43	
Yes	195	17	239	26	696	74		37	3	388	35	705	64		26	2	523	47	581	53	
Rural location							<.001							<.001							<.001
Urban	252	17	315	26	918	74		54	4	509	36	922	64		41	3	680	47	764	53	
Rural	156	37	116	44	145	55		47	11	183	49	187	51		40	10	250	66	127	34	

Author’s analysis of data (2018) from the American Hospital Association (AHA) Information Technology Supplement Survey, AHA Annual survey, and Medicare Final Rule Standardizing File.

Furthermore, the 891 hospitals that indicated using electronic clinical data from the EHR for clinical and organizational evaluation were largely teaching hospitals, with not-for-profit status, part of a system, and located in urban areas. They also had a noticeably lower average HHI with a mean of 0.37 compared with those hospitals that do not use clinical care evaluation capabilities from their EHR (mean, 0.57), a higher number of staffed beds (mean, 296.65), higher case mix index (mean, 1.68), and the highest disproportionate share at 0.04 (Table 2).

**Table 2:** Description of Electronic Health Records (EHRs) Clinical Care, Organizational, or Both Evaluation Capabilities by Continuous Hospital Characteristics, 2018^a^

	Clinical care capabilities				Organizational evaluation capabilities				All capabilities: clinical and organizational evaluation			
	None	1-4	All	*P* value	None	1-3	All	*P* value	None	1-7	All	*P* value
Continuous, mean (SD)												
Herfindahl–Hirschman index	0.50 (0.42)	0.50 (0.40)	0.37 (0.37)	<.001	0.58 (0.41)	0.47 (0.41)	0.38 (0.38)	<.001	0.57 (0.42)	0.47 (0.41)	0.37 (0.37)	<.001
Number of staffed beds	177.93 (168.14)	241.61 (237.45)	293.47 (249.58)	<.001	124.55 (107.25)	228.35 (201.64)	286.83 (257.3)	<.001	107.65 (75.41)	231.88 (218.17)	296.65 (254.18)	<.001
Medicare discharge rate	0.52 (0.15)	0.50 (0.13)	0.51 (0.13)	.018	0.51 (0.16)	0.51 (0.14)	0.51 (0.13)	.538	0.52 (0.17)	0.51 (0.14)	0.51 (0.13)	.471
Medicaid discharge rate	0.22 (0.15)	0.23 (0.12)	0.21 (0.11)	.060	0.22 (0.15)	0.22 (0.13)	0.22 (0.11)	.636	0.21 (0.14)	0.22 (0.14)	0.21 (0.11)	.150
Case mix index	1.52 (0.30)	1.60 (0.30)	1.68 (0.29)	<.001	1.47 (0.32)	1.59 (0.31)	1.67 (0.29)	<.001	1.45 (0.26)	1.59 (0.30)	1.68 (0.29)	<.001
Disproportionate share	0.02 (0.02)	0.03 (0.03)	0.04 (0.04)	<.001	0.02 (0.02)	0.03 (0.03)	0.04 (0.04)	<.001	0.01 (0.01)	0.03 (0.03)	0.04 (0.04)	<.001

Author’s analysis of data (2018) from the American Hospital Association (AHA) Information Technology Supplement Survey, AHA Annual survey, and Medicare Final Rule Standardizing File.

a Standard deviation in brackets; significant relationships is at the *P*<.05 level.

### Evaluation Capabilities: Clinical Care

Model 1 (Table 3) identifies which organizations use their EHRs clinical care evaluation capabilities. When comparing which organizations were using “all” vs those who do not use any of their EHR capabilities, we found that hospitals with a single EHR vendor were more likely (ie, had a greater relative risk) to use all EHR capabilities for clinical care evaluation (adjusted RRR, 2.90; 95% confidence interval [CI], 2.19-3.84) compared with hospitals with best-of-breed EHRs. Also, compared with hospitals located in the West region of the United States, hospitals located in the Northeast region were more likely (RRR, 1.78; 95% CI, 1.1-2.89) to have all EHR capabilities for clinical care evaluation. Hospitals with a higher disproportionate share were more likely (RRR, 1.11; 95% CI, 1.01-1.21) to use all EHR capabilities for clinical care evaluation. Hospitals in rural areas had a reduced likelihood (RRR, 0.58; 95% CI, 0.41-0.83) to use all EHR capabilities for clinical care evaluation compared with urban hospitals. Finally, hospitals with higher Medicaid discharge rates were less likely (RRR, 0.25; 95% CI, 0.07-0.98) to use all EHR capabilities for clinical care evaluation compared with those with lower Medicaid discharge rates.

**Table 3:** Hospital Characteristics Associated With Electronic Health Record (EHR) Clinical Care or Organizational Evaluation Capabilities, 2018: Multinomial Logistic Regression, N=1902^a^

	Model 1: clinical care capabilities		Model 2: organizational evaluation capabilities	
	None vs 1-4	None vs all	None vs 1-3	None vs all
	RRR (95% CI)	RRR (95% CI)	RRR (95% CI)	RRR (95% CI)
Single-vendor EHR (referent: no)	1.15 (0.85-1.55)	2.90^d^ (2.19-3.84)	1.39 (0.87-2.22)	2.63^d^ (1.65-4.21)
Ownership (referent: government)				
For-profit	0.40^c^ (0.22-0.72)	1.21 (0.76-1.92)	0.44^b^ (0.21-0.92)	0.55 (0.27-1.13)
Not-for-profit	1.45 (0.97-2.15)	1.44 (0.99-2.1)	2.36^c^ (1.29-4.32)	1.51 (0.82-2.76)
System member (referent: no)	1.74^d^ (1.26-2.39)	3.24^d^ (2.4-4.37)	2.43^d^ (1.48-3.98)	4.55^d^ (2.78-7.46)
Region (referent: West)				
Midwest	0.71 (0.45-1.14)	1.43 (0.94-2.16)	1.35 (0.65-2.84)	1.88 (0.9-3.91)
South	0.76 (0.49-1.18)	0.94 (0.64-1.39)	1.34 (0.69-2.58)	1.33 (0.69-2.55)
Northeast	1.02 (0.61-1.72)	1.78^b^ (1.1-2.89)	0.53 (0.24-1.17)	0.86 (0.39-1.89)
Teaching (referent: no)	0.81 (0.58-1.12)	1.02 (0.77-1.37)	1.09 (0.66-1.8)	1.11 (0.67-1.83)
Rural location (referent: urban)	1.02 (0.69-1.49)	0.58^c^ (0.41-0.83)	0.76 (0.44-1.34)	0.70 (0.4-1.23)
Herfindahl–Hirschman index	0.73 (0.48-1.11)	0.79 (0.54-1.15)	0.92 (0.49-1.74)	0.77 (0.41-1.44)
Staffed beds	1.00 (1.00-1.00)	1.00 (1.00-1.00)	1.01^c^ (1.00-1.01)	1.01^c^ (1.00-1.01)
Medicare discharge rate	0.25^b^ (0.07-0.96)	0.48^b^ (0.14-1.63)	1.65 (0.27-10.21)	2.39 (0.38-14.92)
Medicaid discharge rate	0.47 (0.11-2.02)	0.25 (0.07-0.98)	0.74 (0.09-6.29)	0.94 (0.11-8.07)
Case mix index	0.86 (0.44-1.69)	1.05 (0.59-1.9)	0.66 (0.26-1.69)	0.92 (0.37-2.31)
Disproportionate share	1.14^b^ (1.03-1.26)	1.11^b^ (1.01-1.21)	0.97 (0.77-1.21)	1.05 (0.84-1.3)

Author’s analysis of data (2018) from the American Hospital Association (AHA) Information Technology Supplement Survey, AHA Annual survey, and Medicare Final Rule Standardizing File.

a Relative risk ratio (RRR) exponentiated coefficients; 95% CI in brackets.

b α<.05.

c α<.01.

d α<.001.

When comparing which organizations are using “some” of the EHR capabilities and those who do not use any (none) of the EHR capabilities, we found that compared with government nonfederal hospitals, for-profit hospitals were less likely (RRR, 0.40; 95% CI, 0.22-0.72) to use some EHR capabilities for clinical care evaluation. We also found that hospitals that are part of a system were more likely (RRR, 1.74; 95% CI, 1.26-2.39) to use some EHR capabilities for clinical care evaluation than hospitals that are not part of a system. Hospitals with higher Medicare discharge rates were less likely (RRR, 0.25; 95% CI, 0.07-0.96) to have some EHR capabilities for clinical care evaluation compared with those with lower Medicare discharge rates. Finally, hospitals with a higher disproportionate share were more likely (RRR, 1.14; 95% CI, 1.03-1.26) to use some EHR capabilities for clinical care evaluation.

### Evaluation Capabilities: Organizational

Model 2 (Table 3) reports the findings of which organizations that are using their EHR’s organizational evaluation capabilities. When comparing which organizations are using “all” of the EHR capabilities and those that do not use any of the EHR capabilities, we found that hospitals with single-vendor solutions were more likely to use all EHR capabilities for organizational evaluation (RRR, 2.63; 95% CI, 1.65-4.21) compared with hospitals with best-of-breed EHRs.

When comparing which organizations are using “some” of the EHR capabilities and those that do not use any of the EHR capabilities, we found that compared with government nonfederal hospitals, for-profit hospitals were less likely (RRR, 0.44; 95% CI, 0.21-0.92) and not-for-profit were more likely (RRR, 2.36; 95% CI, 1.29-4.32) to use some EHR capabilities for organizational evaluation. We also found that hospitals that are part of a system were more likely (RRR, 2.43; 95% CI, 1.48-3.98) to use some EHR capabilities for clinical care evaluation than hospitals that are not part of a system.

### Evaluation Capabilities: Clinical Care and Organizational

Model 3 (Table 4) reports the findings of which organizations are using their EHR’s clinical care and organizational evaluation capabilities. When comparing which organizations used “all” of the EHR capabilities and those that did not use any of the EHR capabilities, we found that hospitals with single-vendor EHRs were more likely to use all the EHR capabilities for clinical care and organizational evaluation (RRR, 3.37; 95% CI, 1.97-5.76) compared with hospitals with best-of-breed EHRs. We also found that compared with hospitals located in the West region of the United States, hospitals located in the Midwest region were more likely (RRR, 2.35; 95% CI, 1.05-5.27) to use all the EHR capabilities for both clinical care and organizational evaluation. Hospitals that are part of a system were more likely (RRR, 6.21; 95% CI, 3.55-10.86) to use all the EHR capabilities for clinical care and organizational evaluation than hospitals that are not part of a system.

**Table 4:** Hospital Characteristics Associated With Electronic Health Record (EHR) Clinical Care and Organizational Evaluation Capabilities, 2018: Multinomial Logistic Regression, N=1902^a^

	Model 3: all capabilities	
	None vs 1-6	None vs all
	RRR (95% CI)	RRR (95% CI)
Single-vendor EHR (referent: no)	1.30 (0.78-2.17)	3.37^d^ (1.97-5.76)
Ownership (referent: government)		
For-profit	0.46 (0.21-1.03)	0.82 (0.36-1.86)
Not-for-profit	2.02^b^ (1.06-3.84)	1.69 (0.86-3.32)
System member (referent: no)	2.73^d^ (1.6-4.67)	6.21^d^ (3.55-10.86)
Region (referent: West)		
Midwest	1.41 (0.64-3.1)	2.35^b^ (1.05-5.27)
South	1.43 (0.71-2.89)	1.53 (0.74-3.15)
Northeast	0.60 (0.26-1.43)	1.17 (0.48-2.84)
Teaching (referent: no)	1.16 (0.67-2.03)	1.25 (0.7-2.2)
Rural location (referent: urban)	0.83 (0.45-1.51)	0.62 (0.33-1.16)
Herfindahl–Hirschman index	0.99 (0.5-1.95)	0.89 (0.44-1.79)
Staffed beds	1.01^c^ (1.00-1.01)	1.01^c^ (1.00-1.01)
Medicare discharge rate	1.32 (0.19-9.05)	1.74 (0.23-12.94)
Medicaid discharge rate	1.20 (0.12-12.4)	0.57 (0.05-6.51)
Case mix index	0.72 (0.27-1.93)	0.90 (0.33-2.45)
Disproportionate share	1.03 (0.78-1.36)	1.09 (0.82-1.44)

Author’s analysis of data (2018) from the American Hospital Association (AHA) Information Technology Supplement Survey, AHA Annual survey, and Medicare Final Rule Standardizing File.

a Relative risk ratio (RRR) exponentiated coefficients; 95% CI in brackets.

b α<.05.

c α<.01.

d α<.001.

When comparing which organizations were using “some” of their EHR capabilities and those that do not use any of the EHR capabilities, we found that compared with government nonfederal hospitals, not-for-profit hospitals were more likely (RRR, 2.02; 95% CI, 1.06-3.84) to use some of the EHR capabilities for both clinical care and organizational evaluation. We also found that hospitals that are part of a system were more likely (RRR, 2.73; 95% CI, 1.60-4.67) to use some of the EHR capabilities for clinical care and organizational evaluation than the hospitals that were not part of a system.

## Discussion

Hospitals and hospital systems vary in the number of EHR systems that they use. Some apply a single-vendor EHR strategy (ie, the use of a single-vendor EHR throughout the hospital) to all the care they provide, whereas others use best-of-breed EHRs (ie, choosing components from different vendors to meet needs).^20^ These strategies create a mixture of outcomes associated with hospital operations, patient safety risks, and costs.^1^^,^^21^, ^22^, ^23^ This study examined the effect of hospital EHR use on hospital operations related to clinical care, organizational process, and both. In alignment with our hypothesis, we found that the hospitals using a single-vendor EHR system care are more likely to have used data from the EHR to support their clinical care evaluation (eg, QI process and monitor patient safety) and organizational management process (eg, measure organizational evaluation and inform strategic planning).

EHRs are real-time, patient-centered records that make information available instantly and securely to authorized users. They are built to share information with other health care providers, such as laboratories and specialists, so they contain information from all the clinicians involved in the patient’s care.^24^^,^^25^ Specifically, the ability to achieve interoperability can help hospitals provide better quality and safer care for patients.^1^ As interoperability increases, EHRs can also improve patient care by creating effective communication for information between parties.^26^ With a single-vendor interoperable EHR system, all hospital departments have ready access to the latest information allowing for a more coordinated, patient-centered care. The standardization of accessible and actionable data from the use of the single-vendor EHR system in a hospital will, in turn, likely increase efficiencies and cost savings for the hospital.^23^

However, it is difficult for clinicians and administrators to make decisions when the data on the patients are dispersed over multiple, noninteroperable EHR systems.^27^ A growing proportion of hospitals use single-vendor EHR systems for inpatient and outpatient services, including 73% of hospitals in this study. This single-vendor EHR strategy is likely to grow because of the potential efficiencies gained through standardization.^6^^,^^8^^,^^23^ As a result, it is not surprising that hospitals using the same vendor EHR system in both their inpatient and outpatient services are more likely to use resultant data to inform their clinical care decisions and organizational management process. Further, the greater use of EHR data because of a single-vendor EHR system in a hospital has been identified to improve hospital performance.^8^^,^^23^^,^^28^^,^^29^ It seems clear that hospitals can and will continue to leverage the EHR data to improve organizational performance. Moreover, those adopting a single-vendor EHR strategy have better capabilities to leverage both clinical and organizational data to inform clinical care and operational performance, likely because of improved interoperablity.^1^^,^^30^

Nevertheless, single-vendor systems are not without relevant problems. First, these systems may reduce customization and physician preferences.^31^^,^^32^ These limitations can contribute to inefficiency and promote problematic workarounds and potential safety errors,^33^ though other studies have indicated safety benefits associated with improved interoperability.^34^ Further, these systems can contribute to physician burnout because of a lack of flexibility and one-size-all approaches to documentation despite differences in practice and care patterns.^35^ Additionally, reliance on a single vendor could lead to monopolistic behavior by the vendor. Over time, organizations with single-vendor solutions may face increasing maintenance, subscription, and upgrade costs, potentially reducing the benefits gained from organizational and clinical performance improvements.^6^

One instance of the potential benefits of a single EHR is observed from the Veterans Health Affairs (VHA) hospitals, which were excluded from this analysis. The VHA system, although currently undergoing an EHR modernization, has been collecting EHRs for decades using a single system.^36^ It has also leveraged its single EHR system to assess and improve quality in various areas. For example, the VHA has leveraged surgical data to create a VHA Surgical Quality Improvement Program, which was subsequently used to develop the private sector version used by the American College of Surgeons.^37^ Similarly, nursing at the VHA has made use of big data to develop the VHA Nursing Outcomes Database to assess a range of variables, including demographic characteristics, financial, nursing-sensitive indicators, and hospital-acquired conditions.^38^^,^^39^ These data sets have promoted QI efforts throughout the VHA system as standardized data fields, and data collection allows for evaluation of quality over time. In turn, this provides an opportunity to compare and evaluate outcomes adequately.^39^, ^40^, ^41^, ^42^

Multiple hospital characteristics were also identified as influential in using the EHR data to inform clinical and organizational practice. For-profit hospitals were less likely to have used the EHR data for their clinical and organizational processes than nonfederal government hospitals. In contrast, nonprofit hospitals are more likely to use the EHR data for organizational processes. This finding builds on previous findings that support differences in the EHR use among hospital ownership types.^43^ Also, compared with independent hospitals, hospitals that belong to a system are more likely to have used electronic clinical data from the EHR in their clinical care and organizational management process. This is important as previous work has identified that hospitals that are part of a system see improvement in clinical care and operational performance compared with independent hospitals.^44^ The current work further suggests that leveraging clinical or organizational evaluation components derived from the EHR provides an opportunity for organizations to understand their current performance better and to leverage that information to meet their mission and improve outcomes.^29^

Finally, this study also reports more variance in hospital characteristics in using the EHR data for clinical care components such as QI process and patient safety than there are for organizational performance components. Although the EHR would be most easily leveraged to improve clinical care, the variation in the care components used paired with the lack of variation for organizational evaluation components indicates that hospitals either have an easier time using this data for organizational evaluation or are more inclined to do so because of financial benefits. Further, the EHR has been described by some as a billing instrument focused more on increasing documentation and less on the clinical outcomes of care.^45^ As such, organizational use of EHRs still appears to be driven mostly by process or volume instead of value, which may be reflected in the divergence of use for clinical care and the greater use of organizational evaluation components.

### Limitation

There are several limitations to this study. First, this is a retrospective cross-sectional study relying on self-reported survey data. As such, the capabilities used may be inaccurately reported or could change over time. More specifically, we cannot determine changes in EHR usage, or if specific EHR components are developed over time. This is an important limitation as capabilities for EHR usage may mature over time despite single-vendor EHR strategies. However, although this may occur, it is, in our opinion, more likely that single-vendor systems provide a stronger likelihood for more robust EHR usage. Next, although we have assessed if the organization uses a single EHR vendor for inpatient and outpatient services, we have not accounted for vendor fragmentation. This is an important limitation as capabilities, and the ability to gather data from separate versions or separate modules within the same EHR vendor product may prohibit the use of clinical care or organizational evaluation components.

## Conclusion

Hospitals continue to seek ways to leverage their EHR data in meaningful ways to impact clinical care, outcomes, and organizational performance. Although most hospitals used their EHR data, usage varied by hospitals with different EHR vendor systems. Hospitals using a single-vendor EHR system were more likely to engage in clinical care processes, organizational evaluation processes, and both. These processes include QI, patient safety, adherence to guidelines, performance profiles, and both unit and organization performance dashboards.

## Potential Competing Interests

The authors report no competing interests.
